# Patterns of genetic liability in individuals treated for depression in different care settings

**DOI:** 10.1192/j.eurpsy.2026.11264

**Published:** 2026-07-31

**Authors:** K. L. Musliner, J. Ø. Sørensen, E. Agerbo, A. J. Schork

**Affiliations:** 1 Department of Affective Disorders, Aarhus University Hospital-Psychiatry; 2 Department of Clinical Medicine, Aarhus University, Aarhus; 3 Institute for Biological Psychiatry, Mental Health Services Copenhagen, Roskilde; 4 Department of Public Health, Aarhus University, Aarhus, Denmark

## Abstract

**Introduction:**

Genetic studies of psychiatric disorders often select cases using information from treatment settings such as primary and secondary care. Although generally acknowledged that these patient populations vary in terms of clinical characteristics such as severity, the potential for differences in their underlying molecular genetic liabilities has not been explored.

**Objectives:**

To examine polygenic profiles of a range of psychiatric phenotypes in individuals treated for depression in primary care only, secondary care only, or both primary and secondary care.

**Methods:**

Data were obtained from the iPSYCH case-cohort study, which includes 141,265 individuals born in Denmark between 1981–2008. Psychiatric diagnoses from secondary care and antidepressant prescriptions from primary care were linked to genetic data via national registers. Individuals who died, emigrated or received a bipolar disorder (BD) or schizophrenia (SCZ) diagnosis before their 10^th^ birthday were excluded. Depression was classified by treatment setting (primary care only, secondary care only, both primary and secondary care). Polygenic scores (PGS) were calculated using LDpred2. Individuals were followed from their 10^th^ birthday until their first depression treatment, emigration, death, or December 31, 2018, whichever came first. Hazard ratios were calculated using weighted Cox regressions with robust standard errors adjusted for birth year, sex and the first 5 principal components.

**Results:**

The sample included 117,305 individuals (49% female), aged 10–35 at follow-up. 12.3% were treated for depression: 8.0% in primary care only, 0.5% in secondary care only and 3.7% in both. Figure 1 shows the hazard ratios for PGSs and depression stratified by treatment setting. PGSs for depression and neuroticism showed the strongest associations with depression with only minor variation across treatment settings. The effect of PGS-depression was strongest for secondary care only (HR=1.49, 95% CI [1.36-1.64]) while the effect of PRS-neuroticism was strongest for treatment in both settings (1.27 [1.22-1.32]). Associations for PGSs for the p-factor, BD and SCZ were notably elevated for secondary care only (p factor: 1.84 [1.60-2.10]; BD: 1.35, [1.22-1.51]; SCZ: 1.31 [1.18-1.46]) relative to the primary care only (p factor: 1.29 [1.24-1.35]; BD: 1.11, [1.07-1.15]; SCZ: 1.12 [1.08-1.16]) and those treated in both settings (p factor: 1.62 [1.54-1.71]; BD: 1.15, [1.10-1.21]; SCZ: 1.18 [1.13-1.24]).

**Image 1:**
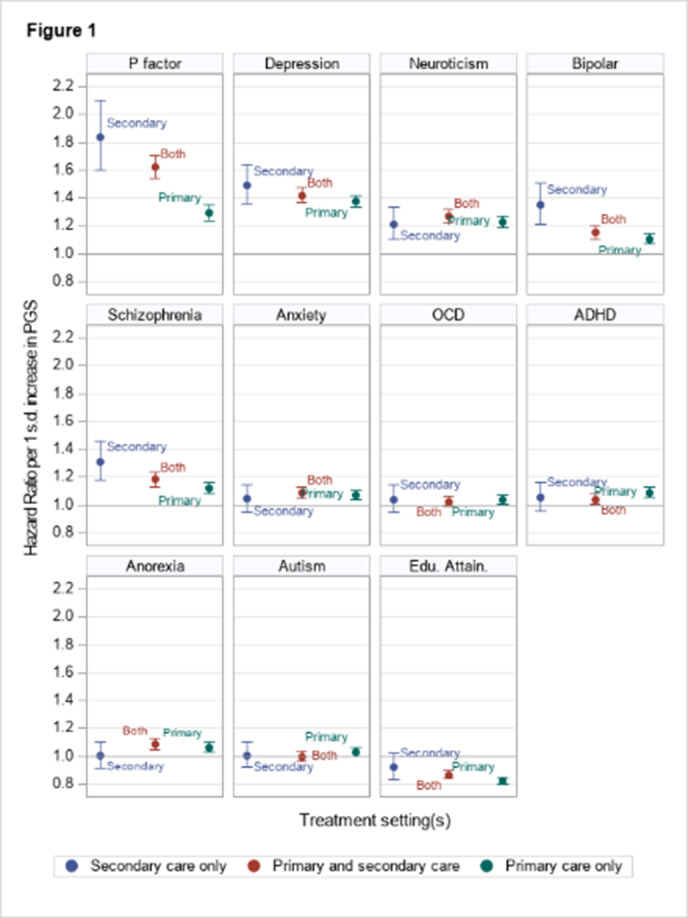
Image 1: Long description.

**Conclusions:**

While individuals treated for depression in different care settings were similar in their polygenic liabilities for depression and neuroticism, there were notable differences between patient groups in terms of polygenic liability for other psychiatric phenotypes, particularly the p-factor, bipolar disorder and schizophrenia. Researchers should consider that case ascertainment methods can have a significant impact on the genetic profile of their sample.

**Disclosure of Interest:**

None Declared

## Long descriptions

### Image 1: Long description

The Y-axis represents Hazard Ratio per 1 s d increase in P G S, ranging from 0.8 to 2.2. The X-axis represents Treatment setting(s). Each panel contains three data points with error bars: blue for Secondary care only, red for Both (Primary and secondary), and green for Primary care only.

* P factor: Secondary is highest at ~1.85, Both at ~1.6, Primary at ~1.3.

* Depression: Secondary at ~1.5, Both at ~1.4, Primary at ~1.38.

* Neuroticism: Secondary at ~1.2, Both at ~1.25, Primary at ~1.22.

* Bipolar: Secondary at ~1.35, Both at ~1.15, Primary at ~1.1.

* Schizophrenia: Secondary at ~1.3, Both at ~1.2, Primary at ~1.15.

* Anxiety: Secondary at ~1.05, Both at ~1.1, Primary at ~1.08.

* O C D: Secondary at ~1.05, Both at ~1.02, Primary at ~1.05.

* A D H D: Secondary at ~1.05, Both at ~1.02, Primary at ~1.1.

* Anorexia: Secondary at ~1.0, Both at ~1.08, Primary at ~1.05.

* Autism: Secondary at ~1.0, Both at ~1.0, Primary at ~1.02.

* Edu. Attain.: Secondary at ~0.9, Both at ~0.85, Primary at ~0.82.

Across most psychiatric conditions, the Hazard Ratio is highest for individuals in Secondary care only, while for Educational Attainment, the ratio is below 1.0 for all settings.

Navigate back to Image 1:.

